# Aberrant functional connectivity of the dorsolateral prefrontal cortex-nucleus accumbens during naturalistic stimulation in adolescent major depressive disorder

**DOI:** 10.3389/fpsyt.2025.1705969

**Published:** 2026-01-05

**Authors:** Yingzhe Zhao, Zhuo Xi, Zhenxiang Zang, Zhi Yang, Jing Liu

**Affiliations:** 1 National Clinical Research Center for Mental Disorders and National Center for Mental Disorders, Beijing Anding Hospital, Capital Medical University, Beijing, China; 2 Harbin First Specialized Hospital, Harbin, China; 3 International & VIP Health Center, Peking University Shenzhen Hospital, Shenzhen, China; 4 Advanced Innovation Center for Human Brain Protection, Capital Medical University, Beijing, China

**Keywords:** depression, dorsolateral prefrontal cortex, functional magnetic resonance imaging, naturalistic stimulation, nucleus accumbens

## Abstract

**Objective:**

This study aimed to examine alterations in the functional connectivity (FC) between the dorsolateral prefrontal cortex (DLPFC) and the nucleus accumbens (NAc) in adolescents with major depressive disorder (MDD) during naturalistic stimulation, and to determine the symptom specificity of this pathway.

**Methods:**

A total of 152 participants were enrolled, including 87 MDD patients and 65 matched healthy controls (HCs). Functional magnetic resonance imaging (fMRI) was conducted during naturalistic video stimulation, and seed-based functional connectivity analysis was performed with the NAc as the seed region. To explore symptom specificity, four factors were derived from the 17-item Hamilton Depression Rating Scale (HAMD-17)—anxiety, depression, insomnia, and somatization— and their associations with DLPFC-NAc functional connectivity were tested in the MDD group.

**Results:**

Our findings demonstrate that adolescents with MDD exhibit significantly higher DLPFC-NAc functional connectivity compared to HCs (P_FWE_ = 0.038). This alteration was positively associated with overall depressive severity, and more specifically with anxiety and somatization dimensions, but not with core depressive or insomnia symptoms.

**Conclusion:**

These findings suggest that DLPFC-NAc hyperconnectivity is a neural substrate of MDD in adolescents and may preferentially contribute to anxiety-related or somatization-related symptomatology rather than depressive mood or sleep disturbance.

## 1 Introduction

Major Depressive Disorder (MDD), recognized as the leading cause of disability worldwide, exhibits a lifetime prevalence of up to 20% and contributes to over one million annual suicides (1). Adolescence constitutes a critical period of psychological and physiological development, among which depression manifests with distinct clinical presentations and profound long-term consequences (2). Although clinical diagnosis relies on the Diagnostic and Statistical Manual of Mental Disorders, Fifth Edition (DSM-5) and Hamilton Depression Rating Scale (HAMD), conventional scale-based assessments face significant limitations, including symptom dimensionality overlap, heterogeneity in treatment response and absence of objective biomarkers (3). These challenges underscore the critical need to elucidate the neurobiological mechanisms underlying depression at the circuit level.

The dorsolateral prefrontal cortex (DLPFC) plays a central role in cognitive control and emotional regulation (4). Dysfunction within the DLPFC has been consistently associated with impaired top–down regulation of negative emotions in depression and anxiety (5). The nucleus accumbens (NAc), a central node of the mesolimbic reward pathway, plays a critical role in reward and motivation (6). Dysregulated dopaminergic signaling and stress-induced synaptic changes in the NAc contribute to maladaptive reward processing and anhedonia in MDD (7–9). Jointly, the functional connectivity (FC) of the DLPFC and NAc plays a vital role in emotional regulation and reward processing in MDD. Resting-state functional magnetic resonance imaging (fMRI) studies have demonstrated that patients with MDD exhibit significantly reduced DLPFC-NAc connectivity, accompanied by widespread abnormalities in static and dynamic FC involving the prefrontal cortex, hippocampus, temporal lobe, and insula, which are significantly correlated with the severity of depressive symptoms (10). From a therapeutic perspective, repetitive transcranial magnetic stimulation (rTMS) targeting the DLPFC-NAc pathway has been found to effectively alleviate anhedonia and mood symptoms, and baseline FC strength within this pathway predicts both antidepressant and anxiolytic treatment responses (11). In adolescents, functional connectivity MRI–guided DLPFC-NAc rTMS has demonstrated significant improvements in anhedonia and depressive symptoms in randomized controlled trials (12, 13). In conclusion, these findings indicate that aberrant DLPFC-NAc FC constitutes a key neural mechanism of MDD and provides a neurobiological basis for precision neuromodulation interventions.

The functional connectivity of DLPFC-NAc is typically estimated using resting-state fMRI. Although resting-state fMRI is widely employed in clinical research due to its high compliance, this approach presents several major challenges. For instance, while resting-state fMRI captures intrinsic neural activity, the high heterogeneity in mental states across individuals and scanning sessions—particularly among patients with affective disorders—introduces significant variability and uncertainty (14). Moreover, adolescents tend to exhibit greater head motion than adults, which further confounds the calculation of functional connectivity (15). Naturalistic stimulation fMRI represents a distinct paradigm in which participants are exposed to standardized stimuli, such as videos (16). This method offers several advantages over resting-state fMRI. First, it elicits more homogeneous brain activity across participants, thereby reducing state-related heterogeneity (17, 18). Second, as participants are engaged in watching a video, their increased attentional load leads to reduced head motion (19). Additionally, naturalistic stimulation allows for more robust estimation of functional connectivity compared to traditional block-based or event-related designs (20), making it particularly suitable for investigating DLPFC-NAc circuitry in adolescent MDD patients.

The current study employed naturalistic fMRI to compare differences in DLPFC-NAc FC between participants with MDD and healthy controls (HCs). We focused on adolescents aged 12-14, a period marked by immature emotional regulation and cognition that increases vulnerability to depression (21). Furthermore, we examined the relationship between DLPFC-NAc connectivity and depression severity and dimensional symptom features. This study aims to identify objective neuroimaging biomarkers and provide a rationale for future naturalistic fMRI-guided rTMS interventions in the treatment of depression. In the present study, we hypothesize that adolescents with MDD will exhibit abnormal DLPFC-NAc functional connectivity compared under naturalistic stimulation. We further aimed to explore the association between the DLPFC-NAc functional connectivity and HAMD-17’s subdimensional factors, providing mechanistic insights for precision neuromodulation.

## 2 Materials and methods

### 2.1 Participants

The current study has been approved by the ethic committee of Harbin First Specialized Hospital. All participants provided written informed consent before participating. MDD patients were diagnosed according to the DSM-5 criteria by a senior pediatric psychiatrist. The child version and parent version of the Mini-International Neuropsychiatric Interview for Children and Adolescents (MINI KID) were used to screen histories of psychiatric disorders. MDD patients with the following inclusion criteria were recruited: (1) age 12 to 14; (2) a consensus diagnosis by two research psychiatrists of major depression according to the DSM-5 on the basis of a Structured Clinical Interview and who have been screened for major depressive disorder by the Brief International Psychiatric Interview, 5th Edition (MINI KID 5.0); (3) an academic degree higher than primary school and capable of completing all study assessments; (4) 14-days medication washout for MDD who were not drug-naïve at current episode; (5) Less than 7 days of medication treatment if for drug-naïve MDD patients. (6) There is no apparent risk of impulse or self-injury at this time; (7) right-handed. The exclusion criteria were: (1) mania or inability to finish study assessments; (2) had no prior history of neurological or psychiatric impairment;(3) a score of 14 or lower on the Hamilton Depression Scale; (4) a history of substance abuse; (5) pregnancy; (6) a history of serious physical disease; (7) excessive head motion (FD > 0.5 mm), and insufficient signal quality in the NAc or orbitofrontal cortex; (8) a contraindication for MRI scans, for instance, with metal implants. As a result, 112 MDD patients and 87 matched- HCs were recruited. A total of 112 MDD patients and 88 healthy controls were initially recruited. After excluding participants with excessive head motion and signal loss in NAc/orbitofrontal gyrus regions, 87 MDD patients and 65 healthy controls were included in the final analysis.

### 2.2 Video stimuli

A silent video clip consisting of 5 public-interest advertisements was used in this study. The length of the advertisements ranged between 1’16’’ to 1’42’’, and the total length of the video clip was 7’50’’ (**Table 1**). We made the video by concatenating materials from four public-interest advertisements and a section of the cartoon “inside-out”. The material selection criteria were: evoking positive or negative emotion, understandable without sound and subtitles, and including scenarios reflecting social stress in school and emotional interactions between children and parents. The audio track and subtitles of the experimental stimulus were removed, considering the influence of language on subjects’ understandings (22). The video and corresponding rating scores are shared at https://github.com/yangzhi-psy/naturalistic_scz.

**Table 1:** Video stimuli content summary.

	Length	Content summary
1	1:16	A love story of two milk bottles. They are produced together, bought together, put in to refrigerator together, but separated when disposing.
2	1:52	Wars harming people and destroying the world are stopped by a kid holding flowers.
3	1:36	A single mother who pretended to be a man to drive a taxi to raise her son.
4	1:34	Transfer girl cries while answering questions in new class.
5	1:42	A hard-working father who suffers from difficulties but pretends to be happy and productive to his daughter.

### 2.3 MRI acquisition

All imaging data were collected with a 3.0 T GE MR750 scanner at the Harbin First Specialized Hospital. Functional MRI scans were acquired using an echo-planar imaging (EPI) sequence (33 axial slices), FOV = 192 mm, matrix= 64×64, slice thickness/gap= 4.0/0.2 mm, TR/TE= 2000/30 ms, flip angle= 90 degree, 300 volumes, duration 10’00”. High-resolution anatomical scans were acquired with a T1-weighted 3D BRAVO sequence (192 sagittal slices, FOV = 256 mm, matrix= 256×256, slice thickness/gap= 1.0/0.0 mm). Functional-fMRI data was acquired in one scanning session during which participants were told to concentrate and watch the video.

### 2.4 Image preprocessing and quality control

The Phipipe data preprocessing protocol was used for the fMRI data (23): (1) the first 5 volumes were discarded to allow MRI signal equilibration; (2) the head movements were realigned over the entire scan; (3) nuisance artifacts such as the 24-parameter head motion time series, the mean signals of the white matter and ventricles, and head motion spikes were regressed out from voxel-wise time series; (4) the 4D data were standardized to a global mean intensity of 10,000. The quality of brain extraction and registration was visually checked; (5) high-pass filtering (> 0.008 Hz). Participants with poor brain registration quality and large head motion were excluded from further analysis. Head motion was evaluated using mean frame-wise displacement (mean FD), and the maximal mean FD was limited to 0.5 mm (**Table 2**).

**Table 2:** Demographic information of the participants.

	MDD (n=87)	HC (n=65)	Statistic
Demographics			
Age (years)	13.47 ± 0.82	12.75 ± 0.77	T (150) = 5.50, p < 0.001
Gender (female/male)	47/40	30/35	χ² (1, N = 152) = 0.63, *p* = 0.43
**Head Motion (mean FD)**	0.16 ± 0.09	0.18 ± 0.12	T=1.50, p=0.14
Depression Severity			
HCs (none)		65	42.76%
Mild	1		0.66%
Moderate	45		29.61%
Severe	41		26.97%

Variables shown in bold represent methodological control variables included to account for potential confounding effects, rather than statistically significant group differences.

While we obtained a distortion-induced signal lose in the orbital frontal area, we applied EPI normalization. Then, we created signal-intensity map for each participant, and further excluded data if the signal-loses overlapped with subgenual cingulate cortex. As a result, fMRI data from 87 MDD patients and 65 HCs were available for further analyses.

### 2.5 Functional connectivity and statistical procedures

We used bilateral NAc from the Harvard-Oxford template as the region-of-interest. The averaged time series from the NAc were extracted and a voxel-wise functional connectivity map was generated using the Pearson’s correlation coefficient. The DLPFC was defined as Brodmman area 9 and 46, as did in previous studies (24). A two-sample t-test was conducted at a statistical threshold of p < 0.05, family-wise error (FWE) corrected at the cluster level within the DLPFC. Subsequently, we explored the relationships between the contrast values from this significant cluster and clinical measures. Linear correlation analyses were performed to examine its association with the total HAMD score. HAMD was further sub-divided into anxiety (item 9,10,11,15,17), depression (item 1,2,3,7,8), insomnia (item 4,5,6) and somatization (item 12,13,16) (25). Building on this framework, our study will examine each dimension separately, aiming to elucidate their specific associations and inform the development of more targeted intervention strategies. The sexual symptom item (item 14) of the HAMD was excluded from the analysis since our participants were adolescents.

## 3 Results

### 3.1 Participant characteristics

We enrolled 152 participants which contains 65 HCs (mean age = 12.75 ± 0.77 years; 46.15% female) and 87 MDD patients (mean age = 13.47 ± 0.82 years; 54.02% female). The severity ranges for the HAMD were applied (26): no depression (0-7), mild depression (8–16), moderate depression (17-23) and severe depression (≥24). Among the 87 participants with depression, 4 participants (2.63%) exhibited mild depression, 45 participants (29.61%) had moderate depression, and 38 participants (25%) had severe depression. The above data indicate a significant proportion of moderate and severe depression cases, underscoring the importance of mental health screening and timely intervention for this age group (**Table 2**).

### 3.2 Group differences in functional connectivity

**Figure 1:**
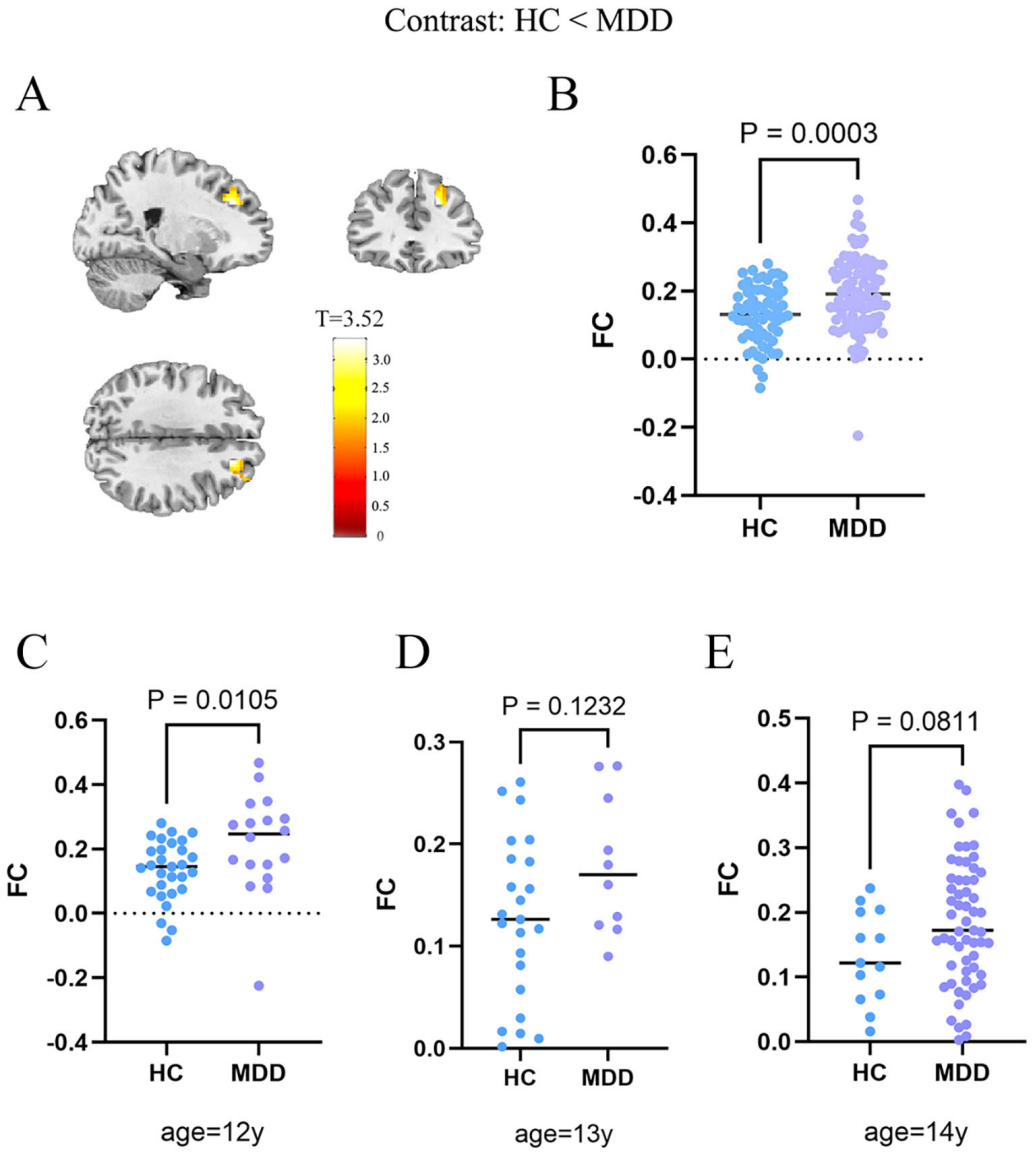
Increased DLPFC-NAc functional connectivity in the MDD. **(A)** Brain regions showing significantly higher DLPFC-NAc functional connectivity in MDD compared with HC. The statistical parametric map is thresholded at the cluster level, and the color bar represents t values. **(B)** Group comparison of extracted functional connectivity values, showing significantly increased DLPFC-NAc connectivity in MDD relative to HC (P_FWE_ = 0.038). Significant cluster: MNI peak coordinate = (48, 51, 6); peak t = 3.52; cluster volume = 123 voxels; effect size (Cohen’s d) = 0.61. **(C)** In the 12-year-old subgroup, adolescents with MDD showed significantly higher DLPFC–NAc functional connectivity than HC (p = 0.0105). **(D)** In the 13-year-old subgroup, the group difference did not reach significance (p = 0.1232). **(E)** In the 14-year-old subgroup, a marginally higher connectivity was observed in MDD compared with HC (p = 0.0811). HC, healthy controls; MDD, major depressive disorder; FC, functional connectivity.

Consistent with the majority of prior work, patients with MDD showed significantly greater FC between the DLPFC and the NAc relative to HC (P_FWE_ = 0.038; **Figure 1**). As a core frontostriatal pathway subserving top-down cognitive control over reward/motivation and affective salience, abnormal DLPFC-NAc coupling may constitute a circuit-level substrate contributing to the pathophysiology of MDD and its symptom expression. To further evaluate the potential effects given the age difference between groups, we conducted a correlation analysis which revealed no significant association between age and DLPFC-NAc functional connectivity (*R* = 0.051, *P* = 0.529). To account for the age difference between groups, we conducted age and gender as covariates of no interest. The group effect on DLPFC-NAc functional connectivity remained significant (p < 0.001, t=3.69). In addition, age-stratified analyses for 12- (p = 0.0105), 13-(p = 0.1232), and 14-year-olds (p = 0.0811) showed a consistent direction of higher connectivity in MDD, with statistical significance in the 12-year subgroup and trend-level differences in the 13- and 14-year subgroups (**Figures 1C, D**). These results suggest that while age contributes to overall variability, it does not account significantly for the DLPFC-NAc difference. Given developmental differences, we re-analyzed the data using a preadolescent-specific NAc parcellation from the Brainnetome atlas (27). The DLPFC–NAc connectivity remained significant (t=3.19, p=0.002; cluster size=60), although the effect did not survive cluster-level correction. This suggests that atlas choice may influence the sensitivity of detecting NAc connectivity in early adolescents, but the direction of the effect remained consistent with the primary analysis.

### 3.3 Symptom-specific connectivity associations

**Figure 2:**
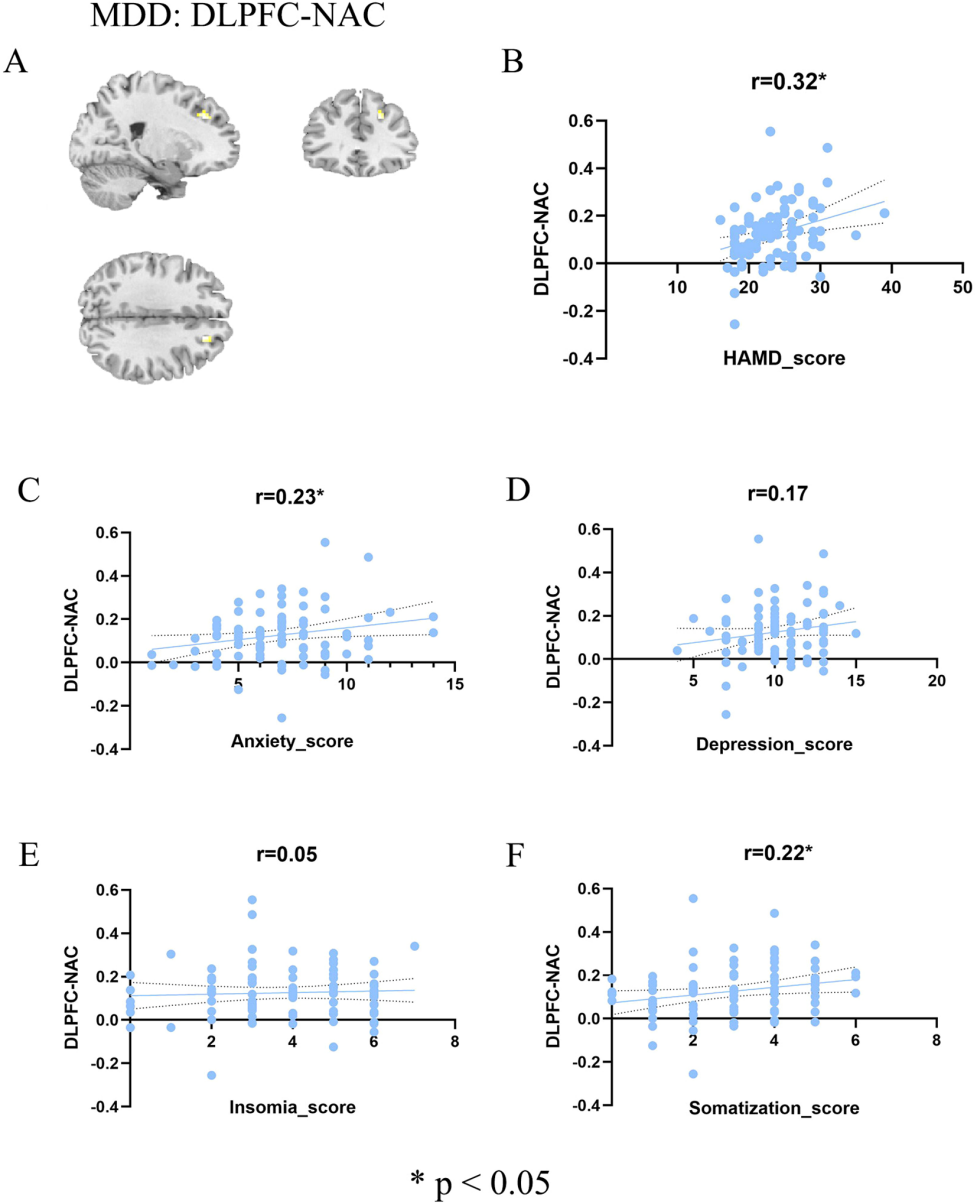
Association between DLPFC-NAc and symptoms in MDD patients. **(A)** Brain regions showing significant DLPFC-NAc functional connectivity in the MDD group (color bar represents t values). **(B–F)** Scatter plots illustrating correlations between DLPFC-NAc functional connectivity and total HAMD score **(B)**, anxiety factor score **(C)**, depression factor score **(D)**, insomnia factor score **(E)**, and somatization factor score **(F)**. Significant positive correlations were observed with total HAMD (r = 0.32, p < 0.05), anxiety (r = 0.23, p < 0.05), and somatization (r = 0.22, p < 0.05), whereas no significant associations were found with depression (r = 0.17, n.s.) or insomnia (r = 0.05, n.s.). HC, healthy controls; MDD, major depressive disorder; FC, functional connectivity; HAMD, Hamilton Depression Rating Scale.

To delineate symptom specificity, we first examined DLPFC-NAc functional connectivity within the MDD group (**Figure 2**). As shown in **Figure 2A**, significant frontostriatal hyperconnectivity was observed. Correlation analyses further revealed a positive association between DLPFC-NAc connectivity and overall HAMD-17 total score (r = 0.32, p = 0.0027; **Figure 2B**). When examining dimensional factors derived from the HAMD-17, connectivity was significantly correlated with anxiety (r = 0.23, p = 0.03; **Figure 2C**) and somatization (r = 0.22, p = 0.04; **Figure 2F**), but not with depression (r = 0.17, p = 0.11; **Figure 2D**) or insomnia (r = 0.05, p = 0.63; **Figure 2E**). These findings indicate that DLPFC-NAc hyperconnectivity in adolescent MDD is preferentially linked to global symptom burden, with particular sensitivity to anxiety and somatic complaints. We applied FDR correction across the four HAMD subdimension correlations. After adjustment, the previously observed associations for Anxiety and Insomnia became non-significant (P-FDR = 0.08), and no subdimensions survived correction.

## 4 Discussion

This study employed naturalistic fMRI to compare functional connectivity between patients with MDD and healthy controls, revealing significantly increased DLPFC-NAc connectivity in the MDD group. Although prior resting-state studies in adults frequently report reduced DLPFC–NAc connectivity in MDD, such findings may not generalize across developmental stages or experimental paradigms. Adolescents aged 12–14 undergo rapid brain maturation, and connectivity patterns during naturalistic emotional stimulation can differ substantially from those observed at rest. Moreover, this enhanced connectivity was positively correlated with the total HAMD score, particularly with its anxiety and somatization factors, but showed no association with depressive symptoms or insomnia. It is important to note that the correlations with HAMD subdimensions were exploratory and did not survive FDR correction. These findings should therefore be interpreted as preliminary and require replication in larger independent cohorts. Therefore, it should be viewed with caution and validated in larger independent samples. These findings provide novel evidence for understanding the neural mechanisms of MDD, highlighting the distinct roles of anxiety and somatization dimensions.

The DLPFC-NAc pathway bridges the central executive network with the reward or motivation system, serving as a critical node at the intersection of cognitive control and emotional processing (6, 28). Prior resting-state studies have reported reduced prefrontal–NAc connectivity in MDD, reflecting diminished reward sensitivity and impaired emotional regulation (9, 29). However, most prior studies were conducted in adults and under resting-state conditions. In contrast, our study focuses on adolescents aged 12–14. Meanwhile, our naturalistic, movie-viewing paradigm requires more attention demanding and social-emotional engagement, which may contribute to the observed hyper connectivity. Importantly, the videos used here were designed to elicit empathic sadness and social-emotional engagement rather than threat or high arousal, making it unlikely that the observed hyperconnectivity is a trait mark. We consider the increased connectivity as a specific marker during emotionally salient naturalistic stimulation and reflects a state-dependent pattern rather than a generalized hyperarousal or a stable trait-level alteration.

These findings suggest that functional connectivity patterns in MDD are not unidirectional but vary according to symptom dimensions and individual differences (30). Notably, hyperconnectivity may represent a state-dependent neural activity pattern (31–33): when anxiety or somatization symptoms are elevated, individuals may exhibit sustained DLPFC-NAc coupling in an attempt to regulate exaggerated responses to threat, bodily discomfort, or uncertainty, whereas in lower-symptom states, connectivity may diminish. This dynamic variability may help explain inconsistencies in previous literature and underscores the importance of investigating symptom fluctuations and state-related changes rather than relying solely on static patient-control comparisons.

A plausible interpretation is that anxiety dimensions are associated with increased connectivity (34), whereas depressive symptoms are more closely linked to decreased connectivity (35). Prior studies on anhedonia have demonstrated that reduced PFC-NAc coupling is strongly related to reward deficits (36). In contrast, our results indicate that anxiety and somatization symptoms are significantly correlated with enhanced DLPFC-NAc coupling. This divergence suggests that abnormalities in DLPFC-NAc connectivity may be symptom-dimension dependent rather than universally characterized by either hyper- or hypo-connectivity (37). In other words, PFC-NAc connectivity should not be considered a unitary biomarker of MDD but rather a dynamic indicator modulated by specific symptom dimensions.

Clinically, these findings bear several implications. First, enhanced DLPFC-NAc connectivity may represent a neural marker of anxiety- and somatization-dominant MDD subtypes, which could aid in more refined, symptom-based classification. Second, this pathway provides a potential target for neuromodulation interventions. Techniques such as rTMS and transcranial direct current stimulation may improve anxiety and somatization symptoms by modulating DLPFC-NAc connectivity. Third, the strength of this functional connection could serve as a neuroimaging predictor of treatment response, contributing to the development of personalized therapeutic strategies. Finally, given that similar patterns of increased connectivity have also been reported in anxiety disorders and somatic symptom-related conditions, our findings raise the possibility that DLPFC-NAc abnormalities may constitute a transdiagnostic mechanism underlying depression-anxiety-somatization comorbidity. Future research including multiple diagnostic groups will be needed to test this hypothesis more directly.

Despite the significance of our findings, there are two limitations should be noted. On the one hand, emotional valence conveyed in naturalistic video materials is inherently subjective, and individuals may differ in their comprehension. Moreover, naturalistic videos often involve rapid shifts in emotional content, whereas the BOLD signal reflects a slow hemodynamic process. These features of naturalistic paradigms may partly influence the observed functional connectivity patterns. On the other hand, due to the limitations of MRI equipment, we were unable to synchronize the transmission of sound information to the subjects. As a result, only visual information was presented, which may reduce the arousal effect of the film. Despite these limitations, our findings provide novel evidence for altered DLPFC–NAc connectivity in adolescents with MDD and highlight the need for future multimodal, longitudinal, and larger-sample studies to validate and extend these results.

## 5 Conclusion

In summary, this study demonstrated that patients with MDD exhibit significantly enhanced DLPFC-NAc functional connectivity compared to healthy controls. Notably, this alteration was positively correlated with the anxiety and somatization factors of the HAMD but showed no association with depressive core symptoms or insomnia. These findings highlight the symptom-dimension specificity of DLPFC-NAc connectivity, suggesting that abnormalities in this pathway may be particularly relevant to anxiety- and somatization-related processes in MDD. Beyond enriching the multidimensional network model of depression, our results also point to DLPFC-NAc connectivity as a promising neural target for individualized interventions. Future studies with larger samples, longitudinal designs, and advanced modeling approaches are warranted to clarify the dynamic role of this circuit in the pathophysiology of MDD.

## Data Availability

The original contributions presented in the study are included in the article/**Supplementary Material**. Further inquiries can be directed to the corresponding author.
