# Supplementary figures and images for: Aberrant functional connectivity of the dorsolateral prefrontal cortex-nucleus accumbens during naturalistic stimulation in adolescent major depressive disorder
Source: Front Psychiatry. 2026 Jan 5;16:1705969. doi: 10.3389/fpsyt.2025.1705969 (PMC12812531; doi:10.3389/fpsyt.2025.1705969)

**Supplementary Materials**

Figure S1.


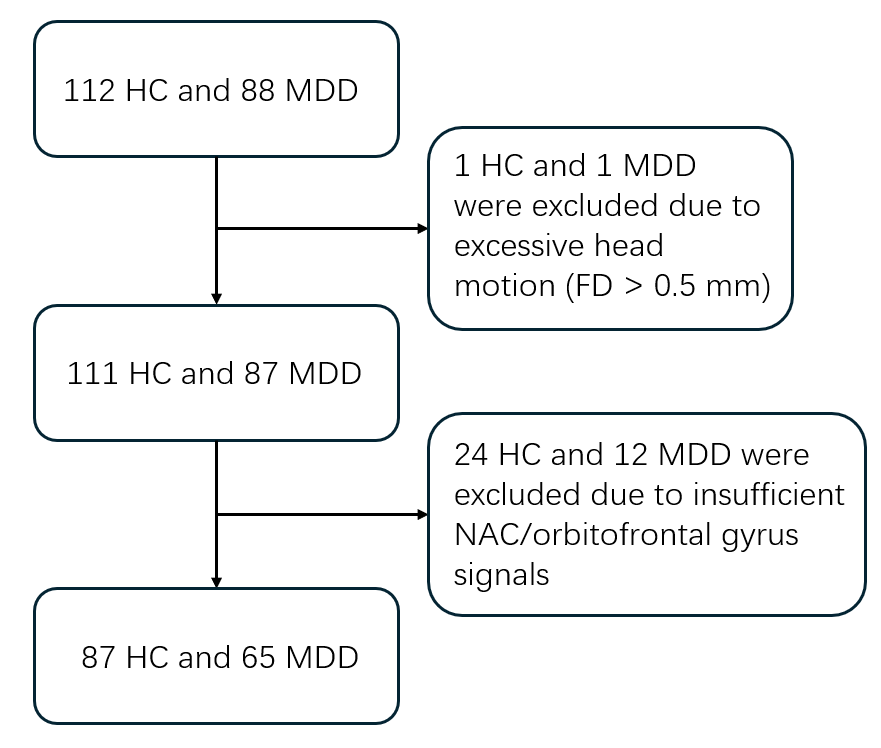


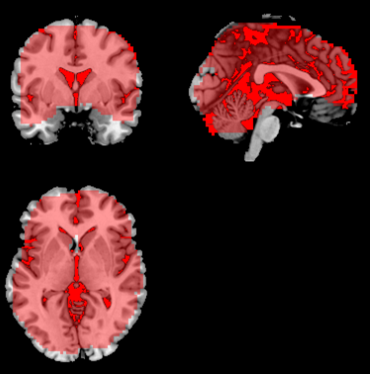

Supplement: Supplementary file 1 [file Table1.docx]
